# To cut or not to cut – that is the question: a comparative analysis of long-term follow-up after complete and incomplete electroconization of the cervix due to high-grade squamous intraepithelial lesion

**DOI:** 10.3389/fonc.2024.1421738

**Published:** 2024-08-15

**Authors:** Barbara E. Suchońska, Małgorzata E. Gajewska, Joanna M. Blok

**Affiliations:** 1st Department of Obstetrics and Gynecology, Medical University of Warsaw, Warsaw, Poland

**Keywords:** cervical cancer, incomplete cervical electroconization, persistent HSIL, positive margins, recurrent HSIL, HPV infection, LEEP/LLETZ, cervical dysplasia

## Abstract

**Introduction:**

Electroconization of the uterine cervix (LEEP/LLETZ) is an appropriate and sufficient procedure for high-grade squamous epithelial lesion – HSIL. Negative margins are considered fundamental for confirming the absence of residual disease. Further management after incomplete excision among women who have not completed their procreative plans is difficult because subsequent cervical procedures may cause issues with carrying a pregnancy to term. Since almost one-third of the untreated patients with HSIL will develop cervical carcinoma, it is essential to balance the desire to radicalize treatment with its obstetric consequences. We compared the further clinical course of the patients after complete and incomplete procedures to observe whether completeness of excision is necessary for a successful outcome. We aim to identify risk factors that influence persistent or recurrent HSIL.

**Methods:**

The study has comprised 781 patients aged 18-85 – the research group was composed of 140 (17.93%) patients after incomplete conization and the control group of 641 (82.17%) patients after the complete one. Patients were scheduled for follow-up examinations every 6 months – including cytology, HPV typing, and colposcopy with tissue sampling. The Chi-square test or Fisher’s exact test was performed as a tool for group comparisons for variables on the qualitative scale. Univariable and multivariable logistic regression models have been used to determine factors associated with the risk of persistent or recurrent HSIL. To evaluate the discriminatory ability of the logistic regression models, the Area Under the Curve (AUC) was calculated.

**Results:**

The statistical analysis results don’t indicate a statistical significance between the frequency of HSIL in groups. HPV infection has increased the risk of persistent/recurrent lesions by 38 times, constituting the most important factor.

**Discussion:**

Close follow-up instead of inconsiderate repeat procedures should be taken under consideration among patients of reproductive age after incomplete conization of the cervix. HPV typing may be an essential method to predict recurrent cervical dysplasia. Promoting HPV typing and vaccination can reduce the number of invasive procedures and improve quality of life and obstetrics outcomes.

## Introduction

Electroconization is an approved treatment for precancerous conditions of the cervix. According to the current ASCCP and SGO guidelines, an excisional treatment (LEEP/LLETZ) is an appropriate and sufficient management of high squamous intraepithelial lesions - HSIL CIN2 and CIN3 (1–5). In the case of carcinoma of the glandular epithelium (AIS), due to its multifocal presence, we more often decide on a radical treatment (3, 6, 7). In patients with HSIL, the use of excisional methods usually allows the therapeutic process to be completed. Negative margins are considered fundamental for confirming the absence of residual disease. The most important prognostic factor is a negative result of the HPV test optimally 6 months after the procedure (1–3, 8–10). In young patients, after an incomplete excisional treatment, when the neoplastic epithelium reaches the incision line or when the incision line runs through the dysplastic epithelium, deciding on further management is much more difficult. It is essential in women who have not completed their procreative plans and in whom subsequent cervical procedures may cause issues with carrying a pregnancy to term (11, 12). Since almost one-third of untreated patients with HSIL CIN3 will develop cervical cancer, it is important to balance the desire to radicalize treatment with its possible far-reaching obstetric consequences (13). The purpose of this research is to analyze the findings of the cytological, colposcopic, and histopathological examinations of the cervix obtained during the long-term follow-up in patients after the electroconization. The aim of the study is to evaluate the risk of persistent and recurrent HSIL after both complete and incomplete procedures and to assess the relevance of repeating the procedure after the initially incomplete conization of the cervix. The study compares the frequency of persistent and recurrent HSIL CIN2+ among patients after the complete and incomplete procedures performed. We aim to identify factors that increase the risk of persistent and recurrent HSIL apart from the status of surgical margins.

## Patients and methods

A total of 781 women aged 18-85 years (median 35.0 [30.0 - 43.0]) who had undergone electroconization of the cervix were included in the research. All women were treated at 1st Department of Obstetrics and Gynecology, Medical University of Warsaw, and then followed up at the Cervical Pathology Outpatient Clinic in 2012-2021. The exclusion criteria were: age of less than 18 or over 85, pregnancy, cervical carcinoma >1A1, diagnosis of other carcinoma, patients lost to follow-up, and immunosuppression therapy lasting 3 years or more. Eventually, the research group comprised 140 (17.93%) patients after the incomplete conization. The determinant of an incomplete procedure was a surgical margin of < 1 mm - described as the presence of dysplastic epithelium that reached the incision line or the passage of the incision line through the abnormal epithelium. Both proximal and distal margins were evaluated. The control group comprised 641 (82.07%) female patients. In 607 (94.70%) women, the conization procedure performed was complete. In 34 patients (5.30%), after the incomplete procedure, a second complete conization was performed within three months, including the patients in the control group. A negative margin was understood as both negative proximal and negative distal margin. The baseline cytology result was abnormal in 629 (80.54%) patients (ASCUS, LSIL, ASC-H, HSIL, AGC). In 152 (19.46%), an excision procedure was performed despite a normal cytology result due to the presence of suspicious macroscopic lesions on the cervix, contact bleeding, or an abnormal colposcopy result. Prior to the procedure, the vaginal portion of the uterine cervix was stained to identify suspicious regions. The cervical conization procedure was performed under brief intravenous general anesthesia with an electric loop, followed by curettage of the cervical canal. The incision borders were marked with ink and the tissue sections have been secured for examination in a 4% formalin solution. Afterwards, the samples were transported to the pathomorphology laboratory. Patients with negative histopathological results in follow-up exams were considered free of persistent and recurrent HSIL CIN2+. Patients were scheduled for follow-up appointments every 6 months, with the follow-up after the procedure for an average of 60 months (min. 6, max. 154). During the follow-up visits, cytology, HPV typing and colposcopy with sampling for histopathology (in patients who showed lesions) were performed. In the past, 175 (22.3%) women had undergone medical procedures treating completely cervical neoplasia (LEEP/LLETZ). The remaining 610 (77.7%) women had no medical history related to this procedure. Prior to the electroconization due to SIL, 11 (1.4%) women were vaccinated against HPV, 15 (1.9%) of them after the procedure. The majority, 759 (97.1%), were not vaccinated at all. Group comparisons for variables on the qualitative scale were performed using the Chi-square test or Fisher’s exact test. For the purpose of factors associated with the risk of persistent or recurrent HSIL CIN2 and CIN3 within the first 2 years after the electroconization, univariable and multivariable logistic regression models were used for the entire research trial and divided into research groups. Results have been presented as odds ratio (OR) and 95% confidence interval (95% CI). To evaluate the discriminatory ability of the logistic regression models, the Area Under the Curve (AUC) was calculated. Analyses were performed using IBM SPSS Statistics 27, and results with p < 0.05 were considered statistically significant. The study protocol was reviewed, and the Bioethics Committee of the Medical University of Warsaw granted the research an exemption from requiring ethics approval. Written informed consent was not required by the Bioethics Committee of the Medical University of Warsaw. The authors confirm that no personally identifiable information is included.

## Results

The incidence of HSIL CIN2+ during the first 2 years after the procedure and throughout follow-up in the research and control group is shown in **Tables 1**, **2**. The results of the Chi-square test of independence don’t indicate a statistical significance between these two groups during two years (χ2(1)= 0.89; p = 0.34) and overall (χ2(1)= 3.26; p = 0.09) follow-up. Throughout follow-up, no HSIL CIN2+ was found in 85.71 to 90.80% of the research group and control group, respectively. Among all HSIL CIN2+ identified (n=79), as many as 73 of them were found during the first two years of follow-up, which equals 92.41% of cases. A total of 68 patients - 6.71% of the control group and 17.86% of the research group - were subjected to repeat complete cervical electroconization due to further findings of HSIL on histopathological examination or suspicious colposcopic imaging. A whole range of dysplastic lesions was noted among the control and research groups, irrespectively of prior lesions, which is shown in **Table 3**. In both groups, the research and control one, univariable logistic regression was carried out to evaluate the risk of persistent or recurrent HSIL CIN2+. The following risk factors for precancerous lesions and cervical cancer were considered: age, medical history of precancerous lesions of the cervix, positive HPV infection status after the procedure for up to 2 years, medical history of other cervical procedures, nicotine addiction, and oral contraceptive administration. Calculations have been conducted for a 2-year follow-up, as shown in **Table 4**. The reference group for the analysis consisted of patients who, among the risk factors listed, had not had the given quality or had the lowest possible value on the ordinal scale (refers to LEEP results). HPV vaccination was excluded from the aforementioned analysis due to the very small number of vaccinated patients and the consequent limitation of obtaining reliable results. In the research group, none of the factors showed a statistically significant increase in the risk of recurrent or persistent HSIL CIN2+ during the first 2 years after the procedure. Among the patients in the control group, statistical significance was demonstrated for the following qualities. For patients ≥ 35 years of age, the risk of recurrent or chronic HSIL CIN2+ within the first 2 years after the procedure is 62% lower than for the patients under 35 years of age [OR = 0.38 (0.22 - 0.68); p <0.001]. For the patients previously treated for dysplastic lesions, the risk of recurrent or chronic HSIL CIN2+ within the first 2 years upon the procedure is 94% higher than for the patients not previously treated for this cause [OR = 1.94 (1.08-3.49); p=0.026]. A statistically significant risk factor for recurrent or chronic HSIL CIN2+ within the first 2 years is a positive HPV infection status after the procedure. Among patients with HPV detected, the risk increases over 38 times compared to the patients with no viral genetic material detected [OR = 38.36 (13.52-108.81); p <0.001]. This is the factor that most increased the risk of HSIL. Among patients who underwent procedures affecting the structure of the cervix, the risk of recurrent or chronic HSIL CIN2+ within the first 2 years after the conization increased by 83% compared to the patients without this quality [OR = 1.83 (1.06-3.17); p = 0.031]. Similarly, nicotine addiction increased this risk over 2 times compared to non-smoking women [OR = 2.11 (1.19-3.74); p = 0.011] and oral contraception by 88% compared to the patients without a medical history for this quality [OR = 1.88 (1.09-3.26); p = 0.024]. We also analyzed the results of multivariable logistic regression to evaluate HSIL CIN2+ risk during a 2-year follow-up after treatment, as shown in **Table 5**. Statistical significance has been indicated for HPV infection after the procedure - the risk of HSIL CIN2+ is then 26.5 times higher than in women who have not been diagnosed with HPV during follow-up [OR = 26.5 (8.73-80.45); p <0.001]. Patients with no dysplastic lesion detected after LEEP/LLETZ have served as a baseline for evaluating the risk of recurrent or persistent HSIL CIN2+ during a 2-year follow-up. Statistical analysis results indicated that patients diagnosed with LSIL on cervical cone histopathological examination presented a 83% lower risk [OR = 0.17 (0.04-0.65); p = 0.010]. Moreover, the results of AUC calculations showed that the model with HPV after the procedure had the highest accuracy (AUC = 0.84; very good).

**Table 1:** Persistent and recurrent HSIL CIN2+ in each group during 2-year follow-up after LLETZ/LEEP.

Persistent/ Recurrent HSIL CIN2+	Control group		Research group		Total		χ²(1)	*p*
	N	%	N	%	N	%		
No	584	91.11%	124	88.57%	708	90.65%	0.89	0.340
Yes	57	8.89%	16	11.43%	73	9.35%		
Total	641	100.00%	140	100.00%	781	100.00%		

HSIL CIN2+, high-grade squamous intraepithelial lesion cervical intraepithelial lesion 2+; LEEP, loop electrosurgical excision procedure; LLETZ, large loop excision of the transformation zone.

**Table 2:** Persistent and recurrent HSIL CIN2+ in individual groups during the entire duration of follow-up after LEEP/LLETZ (electroconization).

Persistent/Recurrent HSIL CIN2+	Control group		Research group		Total		χ²(1)	*p*
	N	%	N	%	N	%		
No	582	90.80%	120	85.71%	702	89.88%	3.26	0.090
Yes	59	9.20%	20	14.29%	79	10.12%		
Total	641	100.00%	140	100.00%	781	100.00%		

HSIL CIN2+, high-grade squamous intraepithelial lesion cervical intraepithelial lesion 2+; LEEP, loop electrosurgical excision procedure; LLETZ, large loop excision of the transformation zone.

**Table 3:** Results of cone histopathological examination after the repeat electroconization in each group.

Result of cervical cone HP examination after subsequent LLETZ/LEEP	Control group		Research group		Total		χ²(3)	*p*
	N	%	N	%	N	%		
No lesions	13	30.23%	5	20.00%	18	26.47%	1.84	0.687
LSIL	14	32.56%	8	32.00%	22	32.35%		
HSIL	15	34.88%	12	48.00%	27	39.71%		
Carcinoma	1	2.33%	0	0.00%	1	1.47%		
Total	43	100.00%	25	100.00%	68	100.00%		

HP, histopathological; HSIL, high-grade squamous intraepithelial lesion; LEEP, loop electrosurgical excision procedure; LLETZ, large loop excision of the transformation zone; LSIL, low grade squamous intraepithelial lesion

**Table 4:** Results of univariable logistic regression for evaluating the risk of persistent or recurrent HSIL CIN2+ in the first 2 years after electroconization in all patients, with the research and control groups identified.

Factors	Total				Control group		Research group		
	OR (95% CI)	p	AUC (95% CI)	OR (95% CI)	p	AUC (95% CI)	OR (95% CI)	p	AUC (95% CI)
AGE (<35^a^ VS. ≥35)	0.52 (0.32 - 0.85)	0.010	0.58 (0.51 - 0.65)	0.38 (0.22 - 0.68)	<0.001	0.62 (0.54 - 0.69)	1.72 (0.60 – 4.92)	0.310	0.57 (0.42 - 0.72)
LEEP – No lesions	^a^	^a^	0.57 (0.50 - 0.64)	^a^	^a^	0.62 (0.55 - 0.69)	^c^	^c^	0.62 (0.55 - 0.69)
LEEP – LSIL	0.52 (0.21 - 1.31)	0.165		0.46 (0.17 - 1.27)	0.134		^a^	^a^	
LEEP – HSIL	1.60 (0.83 - 3.09)	0.160		1.61 (0.82 - 3.17)	0.170		1.92 (0.41 – 9.00)	0.410	
LEEP – Carcinoma	0.68 (0.08 - 5.57)	0.720		^c^	^c^		14.00 (0.62 – 317.38)	0.097	
Medical history of previous dysplatsic lesions^b^	1.94 (1.16 - 3.26)	0.012	0.57 (0.49 - 0.64)	1.94 (1.08 - 3.49)	0.026	0.56 (0.48 - 0.65)	1.88 (0.63 – 5.60)	0.257	0.57 (0.41 - 0.72)
HPV HR prior to the conization procedure^b^	2.16 (0.89 - 5.25)	0.089	0.58 (0.51 - 0.65)	2.18 (0.82 - 5.78)	0.117	0.55 (0.47 - 0.64)	2.00 (0.23 – 17.26)	0.528	0.54 (0.36 - 0.72)
HPV upon the procedure up to 2 years^b^	49.95 (17.81 - 140.07)	<0.001	0.85 (0.81 - 0.89)	38.36 (13.52 - 108.81)	<0.001	0.84 (0.79 - 0.89)	^c^	^c^	–
Age of sexual initiation [YEARS]	0.98 (0.89 - 1.08)	0.689	0.49 (0.43 - 0.56)	0.97 (0.87 - 1.08)	0.630	0.51 (0.43 - 0.58)	1.00 (0.82 – 1.24)	0.966	0.55 (0.41 - 0.69)
Medical history of other cervical procedures^b^	1.76 (1.08 - 2.87)	0.023	0.57 (0.49 - 0.64)	1.83 (1.06 - 3.17)	0.031	0.57 (0.49 - 0.65)	1.59 (0.54 – 4.71)	0.404	0.55 (0.4 - 0.7)
Medical history of transplantation with consequent immunosuppression^b^	1.11 (0.43 - 2.90)	0.827	0.50 (0.43 - 0.57)	0.90 (0.27 - 3.03)	0.868	0.50 (0.42 - 0.58)	1.63 (0.32 – 8.20)	0.554	0.52 (0.37 - 0.68)
Nicotine addiction^b^	1.68 (1.00 - 2.83)	0.049	0.55 (0.48 - 0.62)	2.11 (1.19 - 3.74)	0.011	0.58 (0.49 - 0.66)	0.64 (0.17 – 2.38)	0.501	0.54 (0.39 - 0.68)
Administration of oc^b^	1.93 (1.19 - 3.14)	0.008	0.58 (0.51 - 0.65)	1.88 (1.09 - 3.26)	0.024	0.58 (0.50 - 0.66)	2.18 (0.76 – 6.25)	0.147	0.60 (0.45 - 0.75)

a Reference group.

b Reference group has consisted of patients who have failed to have the quality under consideration.

c No such patients.

CIN, cervical intraepithelial lesion; HPV HR, human papillomavirus high risk; HSIL – high grade squamous intraepithelial lesion; LEEP, loop electrosurgical excision procedure; LSIL, low grade squamous intraepithelial lesion; OC, oral contraception; OR, odds ratio.

**Table 5:** Results of multivariable logistic regression for evaluating the occurrence of persistent and recurrent HSIL CIN2+ up to 2 years in all patients.

Factors	OR	95% CI	p
AGE (<35^a^ VS. ≥35)	0.99	(0.44 – 2.25)	0.987
LEEP – No lesions^a^			
LEEP – LSIL	0.17	(0.04 – 0.65)	0.001
LEEP – HSIL	1.55	(0.60 – 4.01)	0.363
LEEP – Carcinoma	^c^	^c^	^c^
Medical history of previous dysplastic lesions^b^	1.45	(0.37 – 5.76)	0.597
HPV HR prior to the conization procedure^b^	0.91	(0.29 – 2.89)	0.876
HPV upon the procedure up to 2 years^b^	26.50	(8.73 – 80.45)	<0.001
Age of sexual initiation [years] ^b^	1.05	(0.92 – 1.20)	0.473
Medical history of other cervical procedures^b^	0.72	(0.16 – 3.23)	0.666
Medical history of transplantation with consequent immunosuppression^b^	2.17	(0.90 – 5.20)	0.083
Nicotine addiction^b^	1.59	(0.70 – 3.60)	0.266
Administration of OC^b^	1.51	(0.39 – 5.79)	0.548

AUC = 0.89 (0.86 – 0.93)

a Reference group.

b Reference group has consisted of patients who have failed to have the quality under consideration.

c No such patients.

CIN, cervical intraepithelial lesion; HPV HR, Human papillomavirus high risk; HSIL – high-grade squamous intraepithelial lesion; LEEP, loop electrosurgical excision procedure; LSIL, Low-grade squamous intraepithelial lesion; OC, oral contraception; OR, odds ratio.

## Discussion

Our findings are similar to those obtained by the other authors, available in current literature. However, they require a discussion. A significant percentage, as many as 85.71%, of women after incomplete procedure required no further treatment, as no high-grade dysplastic lesions were observed during follow-up examinations. Positive margins were not a factor that constitutes an unfavorable clinical course, as evidenced by the fact that the occurrence of HSIL during follow-up had not been proven significantly different in the two groups, the research and control one. Despite the widely held opinion that there is a higher risk of persistent or recurrent lesions after incomplete conization, radicalization is not recommended today (8, 10, 14–16). However, in our research, 34 patients underwent the repeated conization procedure – among 47.05% of them, no high-grade lesions were detected in the re-amputated cone. Recurrent or persistent HSIL CIN2+ were most common within the first 2 years after the primary procedure. This is consistent with the results published by other authors and the guidelines on which clinicians should base their therapeutic decisions (3, 17). However, it should be borne in mind that women treated for high-grade cervical intraepithelial neoplasia are forever at risk of recurrent dysplastic lesions, as well as other HPV-associated cancers of the anogenital region, including anal cancer (18). The results of univariable logistic regression for the assessment of HSIL occurrence during the first 2 years after the procedure did not allow the distinction of risk factors in the research group. However, an indication of statistically significant risk factors in the control group has been demonstrated. The HPV HR infection after the conization procedure was proven to be the most significant risk factor for recurrent or persistent HSIL CIN2+ - increasing its risk, depending on the statistical tool adopted, from 26.5 (according to multivariable logistic regression) up to 38 times (according to univariable logistic regression), when compared with other factors that increased it to a degree not exceeding 2 times. The model with HPV after the procedure had the highest accuracy according to AUC calculations (0.84). This finding is consistent with data from the literature and current recommendations from scientific associations involved in cervical pathology (3, 9, 10, 16, 18). The presence of HPV infection after the electroconization is an extremely important consideration for rational therapeutic decisions. Therefore, testing for HPV HR should enter the canon of follow-up examinations performed in women after the excision of HPV-associated cervical lesions. The research has found a higher risk of recurrent or persistent HSIL CIN2+ within the first 2 years after the procedure for patients under 35 years of age, indicating the need for careful follow-up in this age group. This may explain the increase in the proportion of young patients diagnosed with invasive cervical cancer (19–21). These results differ from those obtained by other authors, who found a higher risk of progression of precancerous lesions in older women, especially after menopause (10, 14). Multivariable logistic regression analysis demonstrated a higher risk of recurrent or persistent HSIL CIN2+ up to 2 years after the procedure among the patients without confirmed dysplastic lesions in tissue specimens compared to those diagnosed with LSIL. This means that every patient undergoing LEEP/LLETZ, regardless of the result, should be monitored. Some lesions may remain undetected on pathological examination, potentially leading to dysplasia recurrence. According to Kuroki et al. (8), the risk of persistent/recurrent HSIL CIN2+ among patients with a negative result in cervical cone and concomitant cytological HSIL prior to the procedure is comparable to that in patients with confirmed dysplastic cone lesions. In our research, an indicator of incomplete procedure was the presence of dysplastic tissue in margins <1 mm. Although this is a recognized measure of incomplete excision, it should be noted that there are no values for the width of the margins that will guarantee us the absence of persistent/recurrent lesions during the long-term follow-up. Recurrence is not necessarily the result of a lack of completeness of a previously performed procedure but a process that has developed with still active risk factors hence, as already mentioned, therapeutic decisions should not depend solely on the status of the surgical margins. Ouh et al. (9) describe the presence of infection with the same type of HPV as before in 38.7% of patients after the electroconization. On its base, HSIL CIN 2+ can appear in a short period of time, the frequency of which is estimated at almost 15-18% (10, 22).

The research conducted by Bogani et al. aimed to investigate the impact of HPV persistence on the 5-year risk of recurrent HSIL (CIN2+). A total of 545 patients with persistent HPV infection following primary conization were enrolled in the study. The research demonstrated a 7,46% risk of recurrence in patients with HPV persistence at 6 months and a 13,1% risk in those with persistence at 12 months. Surprisingly, HPV infection lasting more than 12 months did not result in a significant rise in the risk of HSIL (CIN2+) recurrence (23).

Despite the existing recommendations concerning screening and the experience we have developed, each patient should be treated individually, according to the best knowledge based on EBM. It appears that regular, sufficiently frequent screening may be a better option than the radicalization of treatment. This is important in women of childbearing age, as the repeat cervical conization performed may ultimately prevent the patient from being able to carry the pregnancy to term. This is also relevant in the context of possible professional liability. There is increasing evidence of a positive effect of adjuvant HPV vaccination after the excisional procedure in patients treated for cervical dysplasia. Di Donato et al. presented a meta-analysis of eleven studies published between 2012 and 2020 and demonstrated that such an approach can reduce the risk of recurrent HSIL CIN2+ by 64%. However, the authors emphasize the lack of studies evaluating the role of the timing of vaccination and the need to clarify it (24). Given the unsatisfactory proportion of vaccinated unaffected population, the perspective of the use of HPV vaccination in patients already diagnosed with cervical dysplasia raises hopes for possible more effective management in this group. A growing number of studies concerning the pros and cons of minimally invasive versus open surgery in early-stage cervical cancer have raised questions about adopting an appropriate approach. In 2020, Bogani et al. presented current evidence on the role of minimally invasive radical hysterectomy in early-stage cervical cancer based on LACC trial and other studies covering the subject. Collected studies revealed worse survival outcomes in patients after minimally invasive surgery in comparison to the open one. Moreover, Bogani et al. emphasize that a fertility-sparing approach, conization plus node dissection, should be considered in early-stage cervical cancer and be included in counseling in women affected by low-volume tumor who express a desire for future childbearing. Controversy regarding the low prevalence of robotic-assisted minimally invasive surgery in LACC trial led to the launch of further research. Recent studies indicate that robotic-assisted surgery may not affect higher rates of negative outcomes. The ongoing phase III prospective Robotic-assisted Approach to Cervical Cancer (RACC) and the trial of Robotic Versus Open Hysterectomy Surgery in Cervix Cancer (ROCC) aim to evaluate the role of such an approach (25). Moreover, in other research in 2022, Bogani et al. conducted a retrospective, multi-institutional study evaluating the impact of the LACC trial on clinical approach and surgery-related morbidity in early-stage cervical cancer. The authors compared 90-day surgery-related outcomes of patients with early-stage cervical cancer before and after the LACC trial. The study highlights a major decrease in patients treated with minimally invasive radical hysterectomy. However, statistical analysis did not indicate a significant difference in number of 90-day and severe (grade 3 or worse) complications (26). As strengths of the study we recognize large sample size, homogeneity of patients included, long-term follow-up, vast combination of risk factors and robust statistical analysis. However, this study has also some limitations. Owing to the retrospective nature of the study, it lacks sample size calculation, which may affect the results. To improve the validity of our conclusions, we have used appropriate statistical methods to analyze existing data. The use of these methods allows us to partially compensate for the lack of a prior sample size calculation and provides the opportunity to obtain valuable conclusions from the available data. Moreover, we carefully monitored the statistical power of the tests performed, which was always more than 0.8. This approach ensured adequate statistical power of the analyses and allowed to obtain reliable results. Another limitation concerns the study being conducted in a single institution. Furthermore, we didn’t obtain information concerning the depth of the conization in histopathological examination results. In the future, it is crucial to emphasize the significance of including this characteristic in the histological description. HPV vaccination has been excluded from uni- and multivariate logistic regression due to the small number of vaccinated patients in a population study, although it might yield interesting results. This factor is also caused by the retrospective nature of the research. The increasing age of patients in their first pregnancy necessitates the establishment of recommendations for management in patients with cervical dysplasia who wish to preserve fertility. A thorough assessment of the clinical situation, taking into account the oncological risk and patients’ will, may enable motherhood with favorable survival rates. It has been shown that numerous risk factors can be easily identified and detected at an early stage if they are introduced as a routine part of follow-up appointments. Some of them are more than crucial, and some are possible to exclude, i.e. those concerning lifestyle habits or HPV status. Further evidence from well-designed prospective studies is warranted to improve knowledge and establish guidelines for the management of cervical dysplasia and early-stage cervical cancer.

## Data Availability

The raw data supporting the conclusions of this article will be made available by the authors, without undue reservation.
